# Management of Severe Developmental Regression in an Autistic Child with a 1q21.3 Microdeletion and Self-Injurious Blindness

**DOI:** 10.1155/2017/7582780

**Published:** 2017-05-25

**Authors:** Cora Cravero, Vincent Guinchat, Jean Xavier, Camille Meunier, Lautaro Diaz, Cyril Mignot, Diane Doummar, Sandra Chantot-Bastaraud, Angèle Consoli, David Cohen

**Affiliations:** ^1^Department of Child and Adolescent Psychiatry, Reference Centre for Rare Psychiatric Diseases, AP-HP, Groupe Hospitalier Pitié-Salpêtrière, Université Pierre et Marie Curie, 47-83 Bd de l'Hôpital, 75013 Paris, France; ^2^Department of Pediatric Neurology, AP-HP, Hôpital Armand-Trousseau, 26 Avenue du Dr. Arnold Netter, 75012 Paris, France; ^3^Department of Genetics, Division of Chromosomal Genetics, AP-HP, Hôpital Armand-Trousseau, 26 Avenue du Dr. Arnold Netter, 75012 Paris, France; ^4^INSERM U669, Maison de Solenn, Paris, France; ^5^Institut des Systèmes Intelligents et Robotiques, CNRS UMR 7222, Université Pierre et Marie Curie, 1 Place Jussieu, 75005 Paris, France

## Abstract

We report the case of a young boy with nonverbal autism and intellectual disability, with a rare de novo 1q21.3 microdeletion. The patient had early and extreme self-injurious behaviours that led to blindness, complicated by severe developmental regression. A significant reduction in the self-injurious behaviours and the recovery of developmental dynamics were attained in a multidisciplinary neurodevelopmental inpatient unit. Improvement was obtained after managing all causes of somatic pains, using opiate blockers and stabilizing the patient's mood. We offered both sensorimotor developmental approach with therapeutic body wrap and specific psychoeducation adapted to his blindness condition for improving his communication abilities.

## 1. Introduction

Cognitive and behavioural regressions among patients with autism spectrum disorder (ASD) are not uncommon. Regression may stem from multiple factors, including environmental problems, psychiatric comorbidity, or painful conditions. Units dedicated to severe acute behavioural states seek to provide both containment and supportive framework for patients. Structured around a medical axis and a psychoeducational axis, they enable a comprehensive developmental assessment and the diagnosis of mixed causes at work in the genesis of regression. They also offer graduated responses to behavioural disorders [1].

Among etiologies of ASD, rare genetic conditions are the most frequently reported causes. Here, we report the clinical course of a 7-year-old boy suffering from ASD and severe intellectual disability (ID) due to 1q21.3 microdeletion. To our knowledge, this is the first case of 1q21.3 microdeletion associated with ASD described in the literature.

## 2. Case Presentation

Our patient was the last child in a family of six, with five healthy sisters. The pregnancy was uneventful, but the child was treasured, being the first boy. Of Congolese origin, he was born in France, from parents who left their country during a civil war. The mother was 41 years old at his birth, and the father 44 years old. There was no inbreeding and no noteworthy family history. The delivery took place at term at 38 weeks of gestation by emergency caesarean section as labour was failing to progress. The birth weight was 2680 g with APGAR scores of 2–6 and ventilation during the first five minutes of life. The patient was breastfed for three months. He was described as “eating little,” regurgitating solid and has always been nourished by fluids and sugary foods (dairy products). Parental concerns started at the age of 18 months. Parents describe a “too quiet” baby, soliciting little, without direct visual contact. When he was 23 months old, he began to walk. He was not toilet trained and had delayed speech development (first words when he was 3 years old and a vocabulary later limited to three words). He showed a strong interest in electric switches and in vibration of the washing machine.

The diagnosis of autism was made at the age of 3 years based on communication and social interaction impairments and restricted, repetitive patterns of behaviour and interests. Behavioural problems were reported early, before the age of 2 years, with intense and repeated mutilations of cheekbones and eyes, culminating in a bilateral blindness at the age of 4 years by intumescent white cataract after numerous surgical complications. Behavioural problems and self-injurious behaviours (SIB) compromised his school attendance. The parents separated when he was five years old.

The patient was hospitalized three times in a child psychiatry unit thereafter with inconclusive antipsychotic treatments. He remained without specialized care. He only received the occasional intervention of associative home help. A major developmental regression was reported at home after the loss of sight: increasingly important attachment to adults and grasping behaviour, loss of walking, increase in SIB, and a diet almost exclusively made up of dairy products.

At the age of 7 years and 7 months, he was admitted in a dedicated neurodevelopmental child psychiatric unit. He was initially prescribed tiapride hydrochloride 40 mg/d, cyamemazine 40 mg/d, paracetamol 600 mg/d, melatonin 6 mg in the evening, hydroxyzine dihydrochloride 25 mg/d when needed, and two ophthalmic eye drops. SIB were prominent and nearly permanent, preventing any interaction: knee blows on his face, punches against his cheekbones, and banging his head against the walls. He did not react to the sound of his name or to simple instructions. He had lost the ability to walk and moved on his back by jerking on the ground or requested to be carried, constantly clinging to adults. Eating at a table was unthinkable. He was spoon-fed, immobilized. Several episodes of regurgitation and vomiting, followed by rhinorrhoea, were observed. These episodes were marked by a refusal of solid oral intake (solid food, medicine), a marked resurgence of crying, irritability, grabbing, and SIB. His intestinal transit was altered, with episodes of diarrhoea (false constipation), encopresis, and coprophagia. He also showed mood fluctuation, alternating between emotional outbursts with bursts of self-stimulatory behaviours (stereotypies) and significant irritability, intolerance to frustration, opposition behaviours, tantrums, and sleeping troubles. The psychiatric evaluation found a severe autistic syndrome (Children Autism Rating Scale (CARS) = 38) and severe ID (Vineland-II development age equivalent to 12 months in areas of communication and social skills and 15 months in autonomy).

## 3. Behavioural and Functional Analysis

The first axis of the neurobehavioural unit is behavioural. Via a functional analysis, we searched for functions (e.g., sensorimotor recruitment, attention seeking, demand avoidance, expression of a refusal, and pain) likely to maintain SIB. Given the extremely high frequency and the severity of SIB at the beginning, the functional analysis was not easy to conduct. It was feasible after managing pain (with protective equipment and medication, see below). Furthermore, nonverbal communication and interpretation of behaviours were more complicated without eye contact. However, we retained the hypotheses of social attention seeking (e.g., increase of SIB when physical contact was lost), demand avoidance (e.g., SIB appearing during educative activities), and sensory stimulation (SIB, in the form of cheekbones hitting, which could occur when the patient was alone, in situations with no interaction). In that case, the use of protective and restrictive equipment permitted reducing the motor response and the sensory stimulation experienced.

As second line, we carried out an analogue assessment, which focused on identification of contingencies maintaining SIB. For example, when his mother visited him, the patient could stay for ten minutes in her arms cuddling her very hard and displaying a few SIB (three episodes of 7, 5, and 3 hits). But when she refused the physical contact with him, SIB were continuous (more than forty times per minute). Also, when his mother got out of the room, the frequency was high and he ended up by going to the nurse who stopped his disruptive behaviour. In that case, mother attention seeking and expression of a refusal were stopped by a social response. A positive social reinforcement proved to be useful. We also observed that SIB were frequent during the transportation procedure to the usual activity room. To help the patient to behave during transfers and to know what to expect, we associated a specific nursery song to each frequent activity. Even so, SIB still occurred during some specific activities. We hypothesized that SIB were a way to escape from too hard tasks and as such we adapted our expectations.

## 4. Medical and Genetic Investigations

**Figure 1:**
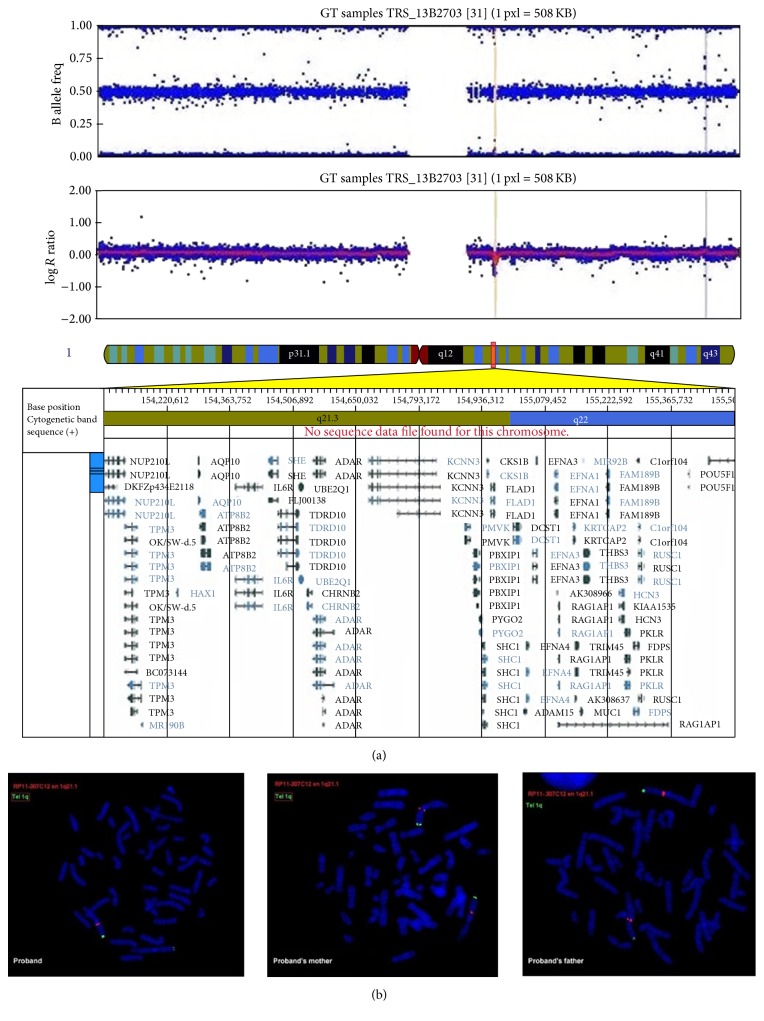
1q21.3 deletion identified in the proband. The deletion contains 65 genes, including 35 genes referenced in the Online Mendelian Inheritance in Man (lower panel) (a). Fluorescence in situ hybridization (probe RP11-307C12, red spots) confirmed that the deletion was present in the proband but not in his parents (b).

**Figure 2:**
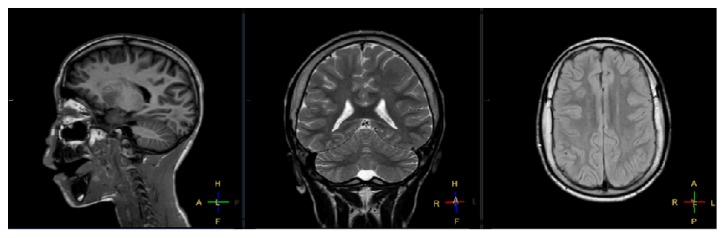
Brain MRI (sagittal, coronal, and axial views) showed a diffuse hyperintensity of supratentorial white matter, a moderate enlargement of the lateral ventricles, a moderate expansion of hemispheric grooves, and a thickening of the cranial vault.

Physically, he was underweight (weight 25% percentile) and had a −0.5 SD size and a body mass index of 14.2 kg/m^2^. In addition to acquired blindness, he presented dysmorphic features: a broad-based flat nose, poor dental status, xerostomia and lanugo, thin legs, genu valgus, genu recurvatum, hyperlaxity, and clinodactyly of 4th and 5th fingers of the right hand and the left and right fifth toe. His head circumference was in the norms. A comparative genomic hybridization array identified a de novo 1.4 Mb microdeletion of chromosome 1q21.3 (chromosomal formula: arr[hg19] 1q21.3q22(154,077,472–155,508,882)x1; Figure 1(a)). Brain MRI found a moderate enlargement of the temporal horns and frontal horns of the lateral ventricles, also associated with a moderate expansion of hemispheric grooves, with thickening of the cranial vault. There was a moderate diffuse hyperintensity of supratentorial white matter and a change of intracranial visual ways associated with atrophy of eyeballs (Figure 2). Echocardiography and abdominal ultrasound were normal.

The possibility of epilepsy was considered, given the high frequency of comorbid epilepsy in autism and ID [2]. The electroencephalogram (EEG) after sleep deprivation showed a somewhat slow wake-up track for his age, with sleep figures and arrangements preserved. Sleep plot showed a predominant intermittent slow activity on the anterior and one or two spikes on temporal. The neuropediatric team did not diagnose epilepsy.

We also searched treatable painful conditions. Upper gastrointestinal endoscopy, conducted as part of postprandial vomiting and a microcytic anemia evoking ulcerative lesions, found* Helicobacter pylori* gastritis and an absence of hiatal hernia. Examination of ears, nose, and throat revealed ear infections and inflammation of the sinus mucosa. The sinus scanner showed a chronic rhinosinusitis, with mucosal thickening and a pansinusien filling. The dental examination revealed numerous cavity lesions. The metabolic balance and blood tests were normal. Under treatment by sodium valproate 750 mg/d, valproate blood concentration was within normal range at 70 mg/L and ammonia blood concentration at 26 *μ*mol/L.

## 5. Treatment

**Figure 3:**
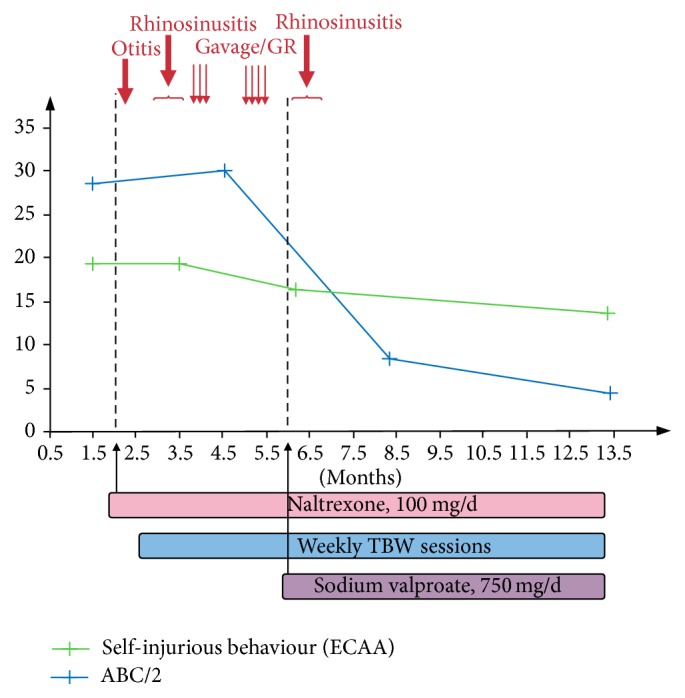
Improving clinical scales during hospitalization (according to the Echelle des Comportements Auto-Agressifs (ECAA) demonstrating the frequency, severity, and duration of self-injurious behaviours, and the Aberrant Behaviour Checklist (ABC)). GR: gastroesophageal reflux; TBW: therapeutic body wrap.

**Figure 4:**
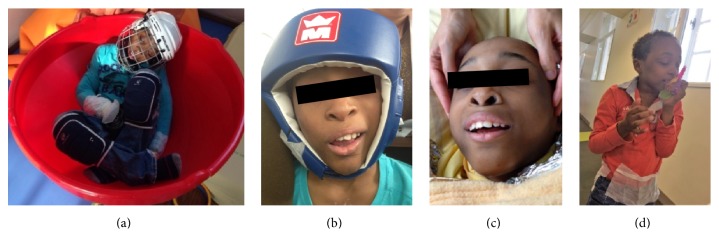
Clinical improvement during hospitalization with decrease of restrictive equipment ((a) to (d)): asleep in the top seat (hard helmet mesh, mittens, and protective knee) (a); wearing soft helmet (b); singing during a therapeutic body wrap session with sensory touch (c); walking without helmet (d).

Significant clinical improvement was observed (Figures 3 and 4) after 13 months of hospitalization involving simultaneously medical, psychoeducational, and sensorimotor treatments. Medical treatment included (1) treatment with opiate blocker (naltrexone 100 mg/d) in order to decrease the endorphin sensation seeking procured by SIB and diminish SIB [3, 4]; (2) prevention of the risk of intestinal obstruction by chronic constipation (frequent in autism [5] and aggravated by antipsychotics [6]) using laxatives, sequential enemas, and a decrease of antipsychotic drug (tiapride hydrochloride and cyamemazine) prescribed to reduce outpatient behavioural problems but without targeting their specific causes; (3) alleviation of physical discomfort by treatment of dental pain (dental care), of ear infections (antibiotics), of rhinosinusitis episodes (nebulized corticosteroids), of* Helicobacter pylori* gastritis (sequential triple antibiotherapy eradication), and of gastro-oesophageal reflux (antireflux medication and split meals) and management of sleeping disorders with melatonin (6 mg in the evening) and continued eye lubrication-hydration by ophthalmic drops; (4) supplements for iron-deficiency anemia; (5) a mood stabilizer treatment with sodium valproate 750 mg/d assuming cyclothymia; (6) support and parental guidance were naturally ensured, whenever possible, throughout the hospitalization.

Psychoeducational treatment involved (1) functional analysis of disruptive behaviours (see aforementioned section); (2) search for reinforcing factors (as music or Tom-Tom to reward and encourage desired behaviours); (3) behavioural therapy targeting challenging behaviours. Initial use of protective equipment (originally mittens, knee pads, and a wire mesh hard helmet, hockey helmet type, and a soft helmet, boxing helmet type) (Figures 4(a) and 4(b)) helped to reduce the likelihood of bodily injury and also reduced the sensory stimulation experienced during and after episodes of SIB. Thereby, the protective equipment served as an extinction mechanism [7]. Given blindness, we proposed to use nursery rhymes and sonorous and tactile objects to improve spatial referencing and transition times between different places and different activities. Positive reinforcement based on reinforcing factors [8] also permitted an improvement in communication skills, decreasing deviant communication and inappropriate behaviours and increasing social initiations; and (4) finally parental mediation, from difficult instruction in the unit and at home, which improved mother-son interactions to fit better the relational distance, nevertheless without succeeding in mobilizing maternal cultural representations and other embedded relational modalities, including exclusive milk feeding.

Psychomotor treatment was based on sensorimotor approach among ASD and adapted to the patient's sensory characteristics [9]. We focused on embodiment, proprioception, and tonic regulation. First, we proposed weekly sessions of therapeutic body wrap (TBW) to help experience a unified body feeling, relaxation, and being held. TBW has been used as an adjuvant treatment in SIB or catatonia associated with autism [10, 11]. Second, we proposed exercises focusing on oral and manual coordination (capture/exploration), front/rear, left/right, and up/down. Third, we developed tailored background and postural supports, designed to promote postural-motor acquisition (back, ventral, feet, and upper limb supports when sitting during meals at table). This support care was based on the patient's sensory characteristics (tactile and olfactory hyposensitivity, auditory and vestibular hypersensitivity, and lack of visual flow). This work on multimodal integration, integration of one's body, tonic fit, postural and motor acquisitions, sensory integration, and untying of fine motor skills played a key role in the possibilities of exploring his environment and in reducing SIB, mostly in the resumption of developmental dynamics, including the most dramatic effect: the resumption of walking and standing up despite blindness.

## 6. Outcome and Patient Perspectives

After a year of hospitalization, the patient was released with a treatment including sodium valproate 750 mg/d, naltrexone 100 mg/d, melatonin 6 mg in the evening, esomeprazole 20 mg/d, lactulose 2 bags/d, and ophthalmic eye drops with a soft helmet. He gained 35 points in the Global Scale Functioning (GSF) and scored 1 (dramatically improved) in the Clinical Global Impression-Improvement scale (CGI-I). His mood was stable, without tantrums or irritability, and he felt pleasure without crippling stereotypes. The SIB were limited to small low intensity fists against his helmet or his cheekbone, occurring from time to time. We attended a developmental recovery, previously unimaginable, with the emergence of social interaction and adaptive capabilities. He resumed walking. During the best of times he wandered half-days without helmet, smiling and exploring his environment using tactile gestures. He enjoyed short walks and riding a tricycle. We attended a little endearing boy in full sensory exploration: receptive to nursery rhymes, laughing during water games, and enjoying the ball swimming pool and “salt paste” workshop. He acquired a verbal language of few words (less than five) and could vocalize rhythmically his favorite nursery rhymes (“Cuckoo Owl” associated with TBW sessions and “Oh, can I say, Mom”). He offered and asked for affection. He could also respond to simple instructions, with a beginning of self-empowerment for daily gestures (washing, meals, and dressing).

## 7. Discussion

We report, to our knowledge, the second case in medical literature of 1q21.3 microdeletion [12] and the first case associated with ASD. We also relate his course. The previous case was a 2.5-year-old girl with microcephaly, epilepsy, ID, and a deletion sharing most of the genes involved in our patient (coordinates converted in hg19 chr1:154,244,969–155,726,479) with no data concerning her emotional development [12]. We found several similarities in the two cases: ID, emergency caesarean, low birth weight, acute fetal distress, difficulty in feeding, weight retardation, dysmorphic features, and above all early SIB. To note, a microdeletion with similar location and size to the previous case and the one in our patient has been reported in genetic database DECIPHER. The phenotype includes delayed language development and ID but no microcephaly nor epilepsy [13]. Our patient had no microcephaly. He was suffering from ASD. If the 1q21.1 region is well known in autism [14], the 1q21.3 region is far less referenced [15]. Though the normal development of children with visual impairment is not well known, blind children developing normally share many traits with sighted children with autism: echolalia, pronoun reversal, delay in the development of symbolic play, SIB, limited theory of mind and pretending play, and “blindism” (stereotyped, repetitive behaviours commonly observed among blind children) [16, 17]. For our patient, clinical evidence for ASD existed before the age of 3 years and before the onset of blindness (when being 4 years old). A key feature of this observation is the precocity of SIB in infancy, leading to dramatic consequences. Contrary to passivity, hypotonia, movement, and emotional or behavioural abnormalities, which are classic signs of parental concerns in ASD [18], SIB are not part of symptoms starting in early childhood. The loss of* CHRNB2* gene, deleted in our patient and in the previously reported one, could possibly be involved in the SIB observed in both of them. Gain-of-function mutations in this gene are responsible for nocturnal frontal lobe epilepsy but no phenotype has been ascribed to allele losses or loss-of-function mutations.* CHRNB2* encodes a subunit of the nicotinic (cholinergic) alpha-4/beta-2 receptor, whose activation has an antinociceptive effect [19]. It is thus possible that* CHRNB2* haploinsufficiency decreases pain sensation and induces SIB.

**Figure 5:**
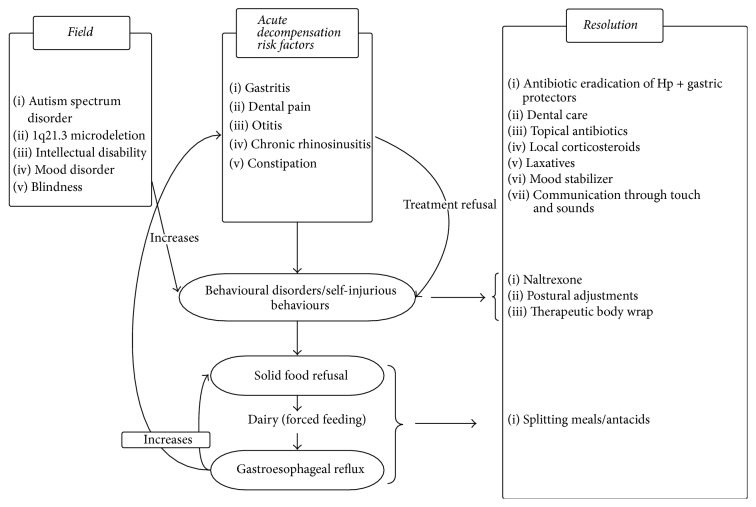
Precipitating factors of acute behavioural crises and implemented therapeutics. Hp:* Helicobacter pylori*.

Besides behavioural dysfunction, we assume that challenging behaviours may have been maintained by somatic pains and pain resulting from SIB. They may have been compounded by the loss of vision and the panic anguish it caused, precipitating the developmental regression. The exclusive and excessive use of dairy products during institutional and during home stays possibly promoted gastroesophageal reflux and perpetuated annoying chronic rhinosinusitis (Figure 5).

Our patient exhibiting severe SIB may have benefited from a trial of naltrexone and TBW. But the operative components of our intervention are not clearly isolated. Behavioural procedures, such as differential reinforcement of alternative behaviour, were not tested in a reversal design format to demonstrate efficacy or lack thereof either. The patient also improved under sodium valproate, both mood stabilizer and broad spectrum antiepileptic. Although the EEG was not conclusive and no seizure was observed during hospitalization nor was it previously reported, we want to emphasize that two genes for epilepsy,* CHRNB2* and* ADAR*, are part of the genes carried by this microdeletion of chromosome 1q21.3 region (Figure 1(a)). To date, epilepsy has been associated with gain of function of both genes (as expressed in a smaller 1q21.3 microduplication of 192 Kb [20]) rather than loss of function. However, given the low number of cases reported so far, no conclusion can be made. A withdrawal of sodium valproate, likewise, would have allowed progress in the clinical reasoning, but in this complex situation, given a positive evolution after prolonged hospitalization, we did not want to risk any deterioration.

The regression was multifactorial, not following the clinical course of a neurodegenerative process. Recuperative capacities are preserved if a multidimensional and integrative management in a neurodevelopmental dedicated unit is provided. Medical care enabled a reduction of symptoms, making the patient available and thus allowing the work of his developmental stakes (occupational therapy and psychoeducational care). We were able to imagine a thread that could have allowed resolution with all the care he received (Figure 5).

## Additional Points

*Key Points*. The 1q21.3 microdeletion seems associated with ID, dysmorphic features, and early SIB and can be a cause of syndromic autism. Severe behavioural problems with SIB associated with ASD, ID, and/or sensory disability may improve in hospitalization in a multidisciplinary neurodevelopmental unit. Treatment of sensorimotor severe autistic pathologies allows children whose sensory and motor characteristics show a sensorimotor integration failure to integrate in a global self-representation of the different stimuli of their environment (auditory, vestibular, proprioceptive, visual, and tactile). In the case of SIB, extreme cases of stereotypies, restraining experiences such as TBW may also be beneficial. The role of the child psychiatrist has been to target the main causes of SIB, including causes of physical pain, and to coordinate specific somatic care, psychiatric care, and the necessary paramedical care.
